# Nutritional detoxification strategies in aquaculture: bioactive feed additives mitigating oxidative stress and xenobiotic toxicity in fish

**DOI:** 10.1007/s10695-026-01729-6

**Published:** 2026-06-26

**Authors:** Mustafa Öz, Enes Üstüner

**Affiliations:** Department of Fisheries and Diseases, Faculty of Veterinary Medicine, Aksaray University, Aksaray, Turkey

**Keywords:** Nutritional detoxification, Xenobiotic toxicity, Oxidative stress, Bioactive feed additives, Nrf2/ARE signaling pathway

## Abstract

The rapid expansion of global aquaculture has increased fish exposure to multiple xenobiotics, including agricultural chemicals, industrial pollutants, and emerging contaminants such as microplastics. Although numerous studies have examined individual toxicants, a critical knowledge gap remains regarding integrative, nutrition-based detoxification strategies that simultaneously mitigate oxidative stress, enhance endogenous detoxification pathways, and reduce dependence on chemical therapeutics. This review addresses the question of how bioactive feed additives can be strategically used to support physiological detoxification mechanisms in fish exposed to complex xenobiotic mixtures. The review synthesizes current evidence on phytogenic compounds, functional amino acids, trace elements, and advanced delivery systems that activate antioxidant defense systems, phase I and phase II detoxification enzymes, and cellular repair processes. Particular emphasis is placed on molecular pathways such as nuclear factor erythroid 2-related factor 2 signaling, species-specific detoxification capacity, and the interaction between nutrition, gut microbiota, and immune function. Comparative evidence across major aquaculture species demonstrates that nutritional detoxification strategies can improve resilience to oxidative damage while contributing to reduced antibiotic use and improved product safety. By integrating mechanistic insights with One Health and sustainability perspectives, this review highlights nutritional detoxification as a scalable and environmentally responsible approach for managing xenobiotic stress in modern aquaculture systems.

## Introduction

The growth of worldwide aquaculture facilities leads to increased toxicant exposure which varies between locations and specific pollutants. The Food and Agriculture Organization (FAO) shows that aquaculture operations continue to expand worldwide but environmental rules and water contamination restrict its development in particular areas. Sustainability challenges in emerging markets are highly pronounced at elevated exposure levels. This vulnerability is primarily driven by distinct feed procurement systems, specialized waste disposal methods, and inconsistent regulatory frameworks (Karim and Almira 2023; Troell et al. 2014). The data show that production growth leads to rising exposure levels but different risk thresholds emerge because of local contaminant patterns and regulatory approaches (Parlakidis et al. 2024; Troell et al. 2014). Climate change profoundly influences bioaccumulation in aquaculture species by altering metabolic rates, food web dynamics, and water chemistry. Consequently, these shifts result in highly divergent contaminant uptake and elimination rates across different species and geographic regions (Froehlich et al. 2018; Teal et al. 2012). The specific local conditions determine regional sustainable aquaculture growth thresholds because areas with strict regulations and limited water availability must limit their expansion (Du et al. 2024; Parlakidis et al. 2024). The research supports a climate-based framework which uses local data to create toxic substance exposure thresholds that follow the natural growth patterns of aquaculture operations (Froehlich et al. 2018; Rosa et al. 2012).

Furthermore, these dynamic climate-induced exposure patterns are heavily compounded by the simultaneous presence of multiple contaminants. Consequently, the combined effects of xenobiotics in aquaculture systems extend far beyond simple additive outcomes. Agricultural runoff, industrial effluents, and microplastics interact to exert severe synergistic toxicities that collectively impair both fish health and growth performance. This chemical co-exposure induces synergy by simultaneously disrupting antioxidant defenses, endocrine signaling, and immune functions. As a consequence, it accelerates the tissue-wide progression of oxidative stress, chronic inflammation, and apoptotic pathways (Diamanti-Kandarakis et al. 2009; Grandjean and Landrigan 2014). The nutritional state of animals affects these interactions because feeds containing nutrients can reduce but not eliminate the ongoing toxic effects (Hoffman and Hennig 2017; Hoffman et al. 2017). Exposome-scale frameworks show that multiple chemicals which occur at the same time produce complex biological effects which cannot be predicted through studies of individual chemicals (Warth et al. 2017; Wei et al. 2022). Tissue-specific pollutant accumulation depends strictly on the chemical properties of the contaminants. Lipophilic organic compounds such as polychlorinated biphenyls (PCBs) and polycyclic aromatic hydrocarbons (PAHs) tend to accumulate predominantly in adipose tissues and the liver. In contrast, heavy metals target gill filaments, the liver, and skeletal muscle, demonstrating highly divergent accumulation profiles across commercial fish species (Hong et al. 2017). The timing of exposure changes based on seasonal input patterns and system maintenance practices which create different exposure patterns that affect crop development throughout the production period (Froehlich et al. 2018; Renzo et al. 2015). The analysis of these interactions needs specific methods which combine systems toxicology with exposome research to study different species and their corresponding environmental areas (Sturla et al. 2014; Whyatt and Perera 1995).

In addition to causing systemic physiological distress, these complex multi-xenobiotic exposures severely restrict the operational boundaries of standard therapeutic interventions. Specifically, the baseline detoxification process in aquatic organisms depends on ATP-binding cassette (ABC) transporters and multixenobiotic resistance (MXR) mechanisms which reduce xenobiotic absorption while increasing their elimination when fish encounter multiple contaminants (Ferreira et al. 2014). The combination of multiple xenobiotics creates an overload for detoxification systems which changes drug pharmacokinetics and makes single-agent chemotherapy less effective for treating internal fish toxins according to reviews about transporter- and metabolism-based resistance in aquatic species (Ferreira et al. 2014). The non-target distribution pattern of many chemotherapeutics along with their limited tissue specificity makes it difficult to remove mixed xenobiotics from the body (Ferreira et al. 2014). The use of antibiotics in detoxification processes and antimicrobial resistance (AMR) creates environmental risks because antibiotic use in aquaculture selects resistant microorganisms which spread their resistance genes into the environment (Hemamalini et al. 2021; Lulijwa et al. 2019). The problem becomes worse because certain areas lack proper regulatory frameworks and policies which results in higher resistance spread and exposure. The use of antibiotics in treatment creates environmental residues that select for resistant microbial communities which makes it difficult to develop detoxification strategies that need these antimicrobial agents (Hemamalini et al. 2021; Lulijwa et al. 2019). The sustainability and marketability of fish products depend on these elements. The use of nutritionally based strategies through targeted non-chemical methods helps reduce residue and resistance issues while supporting eco-industrial goals (Robison-Smith and Cable 2024). The ongoing problem of plastic and persistent xenobiotic contamination forces aquaculture systems to maintain their use of chemical detoxification methods for their operations (Robison-Smith and Cable 2024).

Unlike previous reviews that broadly address individual toxicants or generic nutritional supplements, this review fills a critical gap in the current research landscape by providing a comparative, multi-organ synthesis of targeted bioactive feed additives against complex xenobiotic mixtures. Crucially, we integrate these nutritional strategies into established biomarker-based physiological assessment frameworks such as the Integrated Biomarker Response (IBR) index commonly utilized in modern fish health evaluation thereby bridging molecular detoxification kinetics with standardized field-level diagnostic metrics across diverse aquaculture settings.

The research investigates four main objectives which include the following: (1) The study integrates current understanding of agricultural waste interactions with industrial pollutants and microplastics in aquaculture systems. (2) The research evaluates the performance of detoxification systems equipped with ABC transporters and multixenobiotic resistance pathways against different types of pollutants. (3) The research investigates how different contamination patterns in specific regions affect the development of climate-dependent exposure standards. (4) The research investigates two primary negative outcomes of medical treatments which result in antibiotic resistance development and environmental pollution.

The review supports exposome-scale analytical methods which track contamination patterns through time and show how different species absorb contaminants to create better management systems for aquaculture development and ecosystem protection and safe product production in various environmental settings.

## Conceptual framework: nutritional detoxification in fish

### Definition and physiological basis of nutritional detoxification

The detoxification process in fish and mammals operates through different pathways which include uricolytic and hepatic systems. Fish possess uricolytic enzymes which break down urate into urea, showing they have strong urate degradation abilities that mammals do not possess, thus affecting their detoxification processes and nitrogen waste elimination systems (Andersen et al. 2006; Vigetti et al. 2003). The uricolytic function of this system works together with fish-specific enzyme patterns because different fish groups have different levels of urate oxidase activity which affects their detoxification processes differently than mammals do through their urate salvage pathways (Andersen et al. 2006; Vigetti et al. 2003). The digestive enzyme patterns of fish depend on their feeding behavior and intestinal morphology because carnivorous fish have short intestines containing particular proteolytic enzymes which differ from omnivorous fish. The duration of toxin presence in the intestine depends on enzyme breakdown rates (Hofer and Schiemer 1981). The efficiency of detoxification processes depends on gut microbiota composition because this microbiota affects enzymatic degradation and microbial involvement. The gut microbiota composition depends on dietary patterns and intestinal structural characteristics (Jiao et al. 2023; Li et al. 2024). Notably, differences in enzyme profiles and feed retention times among carnivorous and omnivorous fish may predict varying efficacy of orally administered detoxifying compounds (Miyake et al. 2015; Yang et al. 2024).

### Oxidative stress as a key pathway of xenobiotic-induced toxicity

The physiological consequences of xenobiotic exposure in fish are primarily driven by the excessive generation of reactive oxygen species (ROS), which rapidly overwhelm endogenous antioxidant defenses. This imbalance induces widespread oxidative damage to cellular lipids, proteins, and DNA, primarily through the disruption of key protective enzymes specifically superoxide dismutase (SOD), catalase (CAT), glutathione peroxidase (GPx), and glutathione S-transferase (GST) alongside the acute depletion of cellular glutathione (GSH). While acute exposure often halts enzyme production, sub-lethal chemical pressures can paradoxically upregulate or dysregulate these antioxidant enzymes as a compensatory survival response across gill, liver, and kidney tissues (Al‐Ghanim et al. 2020; Balali‐Mood et al. 2021; Öz et al. 2024a, b; Öz et al. 2024a; Öz et al. 2026a, b; Öz and Üstüner 2026a; Öz et al. 2025a, b; Siddique et al. 2020). This tissue-level disruption directly translates into systemic health and performance declines. Elevated ROS levels and elevated lipid peroxidation markers, such as malondialdehyde (MDA) and 8-hydroxydeoxyguanosine (8-OHdG), shift the intracellular GSH/GSSG ratio toward an oxidized state, directly correlating with decreased growth rates, impaired feed efficiency, and structural tissue pathologies (Hassan et al. 2022; Lazado et al. 2021; Li et al. 2022; Öz, 2025). Consequently, tracking these specific antioxidant responses serves as a highly reliable diagnostic matrix for toxicant tolerance prediction and baseline damage assessment. Understanding these tissue-specific oxidative thresholds allows for the establishment of precise risk assessment frameworks, predictable severity limits, and optimized nutritional mitigation strategies crucial for maintaining aquaculture health and biomass stability under xenobiotic duress (Çelik et al. 2024; Esen et al. 2025; Gümüş et al. 2026; Tok et al. 2025).

### Mechanistic targets of nutritional detoxifiers: antioxidant defense, immune modulation, cellular repair

**Fig. 1:**
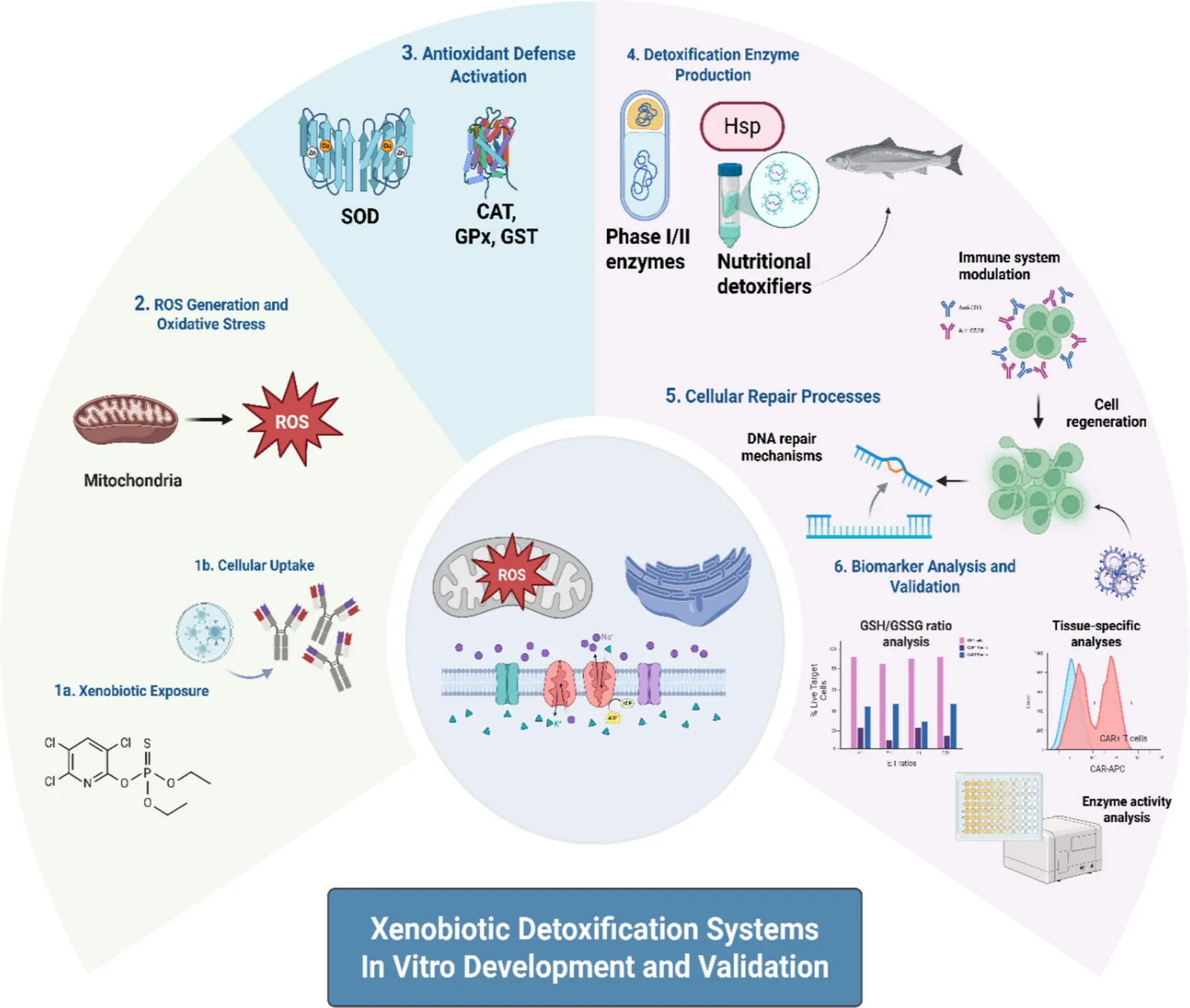
Schematic representation of xenobiotic detoxification mechanisms in fish cells in vitro development and validation platform. The process includes: (1a) xenobiotic exposure to pesticides and heavy metals through various routes, (1b) cellular uptake via membrane transport, (2) ROS generation and oxidative stress in mitochondria, (3) antioxidant defense activation including SOD, CAT, GPx, and GST enzymes with GSH cycle, (4) detoxification enzyme production including phase I/II enzymes and HSP proteins, (5) cellular repair processes involving DNA repair and immune modulation, and (6) biomarker analysis and validation through enzyme activity measurements and tissue-specific analyses in gills, liver, and kidney

To systematically counteract these tissue-specific oxidative thresholds, functional dietary interventions must be deployed to engage specific molecular targets. Across various chemical classes, nutritional detoxifiers achieve systemic cytoprotection by precisely coordinating a tri-phasic detoxification cascade spanning oxidation (phase I), conjugation (phase II), and excretion/efflux (phase III). Crucially, xenobiotic-induced ROS signaling serves as a direct negative regulator of phase I cytochrome P450 (CYP450) activity. Elevated intracellular ROS downregulates CYP450 expression through oxidative modifications of its catalytic heme moiety and molecular crosstalk that inhibits downstream transcriptional pathways. Bioactive feed additives successfully resolve this roadblock by neutralizing excess ROS, thereby shielding phase I oxidation processes from oxidative inhibition. For example, transitioning fish diets toward optimized lipid or vegetable oil formulations fundamentally modulates these gastrointestinal profiles, preserving phase I CYP450 metabolic capacity while concurrently upregulating phase II conjugating enzymes and Phase III ATP-binding cassette transporters to ensure unhindered contaminant clearance (Morais et al. 2012). Small-molecule antioxidants including N-acetylcysteine (NAC) precursors in hepatocytes and other cells increase glutathione production to defend against lipid peroxidation and ROS-induced damage which demonstrates how detoxifier compounds help maintain redox homeostasis (Ahmadian et al. 2017; Olsvik et al. 2007). The detoxification process triggers retrograde signaling pathways which operate with transcriptional networks to maintain xenobiotic sensing while connecting detoxification genes to cellular stress response mechanisms across species (D’Alessandro et al. 2018; Dott et al. 2018). These antioxidant pathways work in close synergy with immune and cellular repair networks. By stabilizing the intracellular redox buffer, these defense systems shield immune cells from excessive oxidative stress, thereby maintaining critical signaling cascades and supporting structural DNA repair mechanisms under chemical pressure (Padmini and Narayanan 2017; Petřivalský et al. 1997). The cellular defense system of phase II responses through glutathione conjugation and multixenobiotic resistance transporters (MRTs) works together with heat shock and DNA repair mechanisms to protect cells from xenobiotic damage through increased expression of heat shock proteins (HSPs) and repair genes in detoxifier-treated systems (Dettleff et al. 2022; Padmini and Narayanan 2017). The detoxification systems of plants and animals show identical patterns because their antioxidant defenses enable the immune system to prepare and tissue repair which results in enhanced production results when dealing with toxic substances (D’Alessandro et al. 2018; Kim et al. 2025; Petřivalský et al. 1997). Figure 1 demonstrates the complete xenobiotic detoxification process.

### Integration into One Health and sustainable aquaculture paradigms

Modern aquaculture operations achieve environmental sustainability through nutritional detoxification because they reduce chemical therapeutic use and decrease pollution while improving feed utilization. The metabolic process of xenobiotics through phase I and phase II enzymes becomes more efficient because nutritional detoxifiers enhance redox defenses which minimizes toxic compound release into the environment and maximizes resource utilization in production systems (Morais et al. 2012; Olsvik et al. 2007). The strategies work through fish detoxification systems that become more efficient when they receive plant-based or optimized lipid formulations which allow contaminants to transform naturally in their environment thus lowering effluent toxicity and improving ecosystem health (Olsvik et al. 2007). The use of antioxidant-rich feeds containing N-acetylcysteine (NAC) precursors and selenium supplements helps protect fish growth by reducing lipid peroxidation while maintaining sustainable production levels without requiring additional chemical additives (Hassan et al. 2022; Olsvik et al. 2007). The One Health-oriented detoxification approach helps people handle waste properly by reducing the amount of waste products that enter the environment through effluents (Lagutkina et al. 2023). Human health depends on two essential factors which involve protecting tissue structure and stopping toxic substances from accumulating in food-ready tissues. The implementation of detoxification-enhanced aquaculture methods decreases toxic residue levels while safeguarding food quality to fulfill consumer safety requirements (M. A. O. Dawood et al. 2022a, b; Li et al. 2022). The implementation of One Health principles through detox gene surveillance and food safety testing enables the creation of unified protection plans which defend animals and their habitats and maintain public health (Jiang et al. 2023; Turner et al. 2024). In this context, transitioning to microalgal biorefineries represents a scalable paradigm to decouple modern aquaculture expansion from finite marine resources while maintaining product quality (Öz and Üstüner 2026b).

## Mechanisms of action of bioactive detoxifiers

### Antioxidant enzyme regulation

Accumulating research indicates that bioactive compounds regulate antioxidant enzyme activity through distinct profiles dictated by tissue origin, species identity, and environmental variables. Mechanistically, the nuclear factor erythroid 2–related factor 2 (Nrf2)/ARE signaling cascade and its molecular crosstalk with mitogen-activated protein kinase (MAPK) pathways function as the primary regulatory networks governing this response.

This enzymatic regulation exhibits high tissue- and environment-specific dependency. Dietary phytochemicals and lipophilic antioxidants like astaxanthin strongly trigger the transcriptional upregulation of SOD, CAT, GPx, and GST across hyper-exposed gill filaments, metabolic liver hepatocytes, and white muscle tissues. Because gills operate under constant oxygen-rich conditions, they experience heightened baseline ROS generation, which frequently results in more robust, inducible antioxidant responses compared to internal tissues when stimulated by identical dietary compounds (Moezzi et al. 2025; Shastak and Pelletier 2023). This protective response is initiated by specific upstream molecular triggers that modify the Kelch-like ECH-associated protein 1 (Keap1)-Nrf2 repressor complex. Dietary bioactives and electrophilic phytochemicals directly interact with highly sensitive cysteine residues specifically Cys151, Cys273, and Cys288 on the sensor protein Keap1. This interaction induces conformational changes that disrupt Keap1-mediated ubiquitination, preventing Nrf2 degradation. Concurrently, specific phytogenics activate upstream intracellular kinase pathways, including protein kinase C (PKC) and MAPKs, which phosphorylate Nrf2 at critical serine and threonine residues. Both upstream mechanisms facilitate the stabilization, accumulation, and subsequent nuclear translocation of Nrf2 to bind antioxidant responsive elements (ARE) within gene promoters (Dinkova‐Kostova and Talalay 2008; Hayes et al. 2010). Crucially, the practical implementation of these bioactives must account for temperature- and oxygen-dependent enzyme kinetics in poikilothermic teleosts, which dictate both transcriptional onset and overall protein turnover. While the Nrf2/ARE signaling cascade initiates within hours of initial exposure, prolonged or excessive dosing can lead to counterproductive pro-oxidant adaptations or cellular growth disruption (Chang et al. 2018; Dinkova-Kostova and Talalay 2008; Hayes et al. 2010). Accurate bioactive dosing schedules must therefore be synchronized with target enzyme half-lives and specific transcriptional peaks to secure true cytoprotective outcomes. However, a major limitation of this transcriptional mechanism is the remarkably narrow therapeutic window of many phytochemical triggers; any prolonged over-supplementation can inadvertently shift their role from antioxidants into pro-oxidants, paradoxically accelerating cellular damage. In aquaculture practice, this indicates that prophylactic administration of Nrf2-activating phytochemicals must be carefully timed prior to anticipated seasonal toxicant surges (such as agricultural runoff episodes), rather than used as a reactionary post-exposure treatment when enzymatic capacities are already exhausted.

### Modulation of lipid peroxidation and redox homeostasis

Ultimately, optimizing the precise kinetics of Nrf2-mediated antioxidant enzyme induction serves a critical downstream purpose: the direct suppression of membrane-level degradation. Consequently, the mechanism of lipid peroxidation inhibition protects fish cells from toxic substances by maintaining membrane structure and preventing lipid damage which decreases ROS production and protects mitochondrial function. The protective effects on redox homeostasis trigger antioxidant defense system activation through cytoprotective pathways. The antioxidant properties create stable glutathione (GSH) levels and decreased lipid peroxidation byproducts which show improved redox status after treatment (Poopal et al. 2017). The GSH/glutathione disulfide (GSSG) equilibrium in redox homeostasis depends on nutritional detoxifiers which supply substrates for glutathione production and neutralize reactive oxygen species (ROS); research indicates that antioxidant supplements and feeds enhance cellular resistance to toxins by increasing reduced glutathione levels and creating beneficial GSH/GSSG ratios (Poopal et al. 2017). Research findings about antioxidant supplement effects on GSH/GSSG ratios show inconsistent results between different studies. The assessment of total antioxidant status (TAS) and total oxidative status (TOS) in fish detoxification research requires established methods which use proven colorimetric or chemiluminescent assays with uniform sample preparation and calibration and reference controls to measure TAS and TOS levels for detoxification effectiveness evaluation. The process of standardizing data between studies continues to be essential because it allows researchers to compare results between different fish species and different exposure conditions (Caldarone et al. 2006; Poopal et al. 2017). In aquaculture practice, this indicates that tracking the systemic total antioxidant versus oxidative status (TAS/TOS) ratio provides farm managers with a sensitive diagnostic matrix to evaluate the protective efficacy of dietary redox-stabilizers against rancid feed fats or waterborne organic pollutants before macro-level production and growth losses manifest.

### Chelation and binding of xenobiotics and heavy metals

While maintaining intracellular redox homeostasis effectively mitigates organic chemical contaminants, non-biodegradable heavy metal toxicity demands a distinct, direct interceptive strategy. To address this, heavy metal chelation in fish feed requires both synthetic and natural chelators to control metal availability and their tissue distribution in fish. The metal excretion process from kidneys becomes more effective through hydrophilic chelators including meso-2,3-dimercaptosuccinic acid, but these compounds fail to reach metals inside cells which reduces their ability to affect metal distribution in tissues unless used with additional chelators or antioxidants (Flora and Pachauri 2010; Flora et al. 2013). The body uses complex formation and targeted intracellular delivery to remove metals through its natural chelators which include polysaccharide-bound ligands and thiourea derivatives and phytochelatins and glutathione and metallothioneins that show specific metal binding properties. Research shows these compounds work to decrease oxidative stress while enhancing body detoxification systems (Iddrisu et al. 2024; Labbé et al. 1993; Sears 2013; Vatamaniuk et al. 2000). The selectivity and affinity of these chelating agents are strictly dictated by the electronic configuration of the target heavy metals and the specific coordinate chemistry of the ligand’s functional groups. According to the Hard and Soft Acids and Bases (HSAB) principle, soft Lewis acids like cadmium (Cd^2^⁺) and mercury (Hg^2^⁺) display an exceptionally high binding affinity for soft electron donors. Specifically, the sulfhydryl/thiol (SH) groups present in thiourea derivatives, glutathione, and metallothioneins form stable, highly insoluble covalent bonds with these soft metals. In contrast, borderline or hard toxic metals like lead (Pb^2^⁺) exhibit preferential coordination with oxygen- and nitrogen-donating functional groups. Carboxyl (COOH), hydroxyl (OH), and amino (NH₂) groups found in polysaccharide-bound ligands stabilize these metals by forming multi-dentate ring structures, or chelates, that significantly accelerate their chemical sequestration. Consequently, these structural configurations directly determine the overall detoxification efficiency (Camci-Unal and Pohl 2009; Haas and Franz 2009; Schalk et al. 2011; Vatamaniuk et al. 2000). Successful heavy metal clearance depends on selecting ligands whose active functional groups chemically match the target contaminant, optimizing detoxification across both circulatory and intracellular compartments (Camci-Unal and Pohl 2009; Flora and Pachauri 2010; Sears 2013; Vatamaniuk et al. 2000). In aquaculture practice, this indicates that when farming in historical mining or industrial zones contaminated with heavy metals like cadmium or mercury, incorporating natural polysaccharide-bound ligands or thiourea derivatives into commercial diets directly intercepts waterborne metallic toxicants within the digestive lumen. This chemical entrapment prevents their systemic assimilation and subsequent hazardous deposition into food-ready fish fillets.

### Hepatoprotective and nephroprotective functions

Therefore, by successfully binding and accelerating the clearance of these systemic toxicants from blood and cellular compartments, functional additives directly shield the primary metabolic and excretory organs. As a result, The Nrf2/ARE signaling cascade serves as the primary molecular target for hepatoprotective nutrients, driving the upregulation of phase II conjugating enzymes and efflux transporters to enhance hepatocyte resilience against organic toxicants (Bryan et al. 2013; Lee et al. 2019; Shen et al. 2014). Concurrently, these bioactive compounds exert profound nephroprotective functions, maintaining operational renal waste elimination systems and stabilizing serum creatinine, urea, and uric acid profiles via conserved vertebrate defense pathways (Jigorel et al. 2005; Lee et al. 2019; Shen et al. 2014). At the tissue level, these synchronized molecular responses manifest as significant histopathological recoveries. This macro-structural preservation—characterized by the suppression of focal hepatic necrosis, maintenance of intact sinusoidal architectures, and mitigation of renal tubular dilation—is directly governed by molecular pathways controlling apoptosis and compensatory tissue regeneration. Specifically, bioactive detoxifiers restore the intracellular Bcl-2/Bax ratio, effectively suppressing the activation of downstream executioner enzymes like Caspase-3 to halt xenobiotic-induced programmed cell death. Concurrently, these additives accelerate parenchymal organ repair by upregulating key tissue regeneration markers, including proliferating cell nuclear antigen (PCNA) and specific cyclin-dependent kinases. These factors drive active cell cycle progression, replacing damaged hepatocytes and nephrons to restore full organ functionality (Bryan et al. 2013; Lee et al. 2019; Liao et al. 2024; Lim et al. 2025). In aquaculture practice, this indicates that functional feed additives targeting organ-level structural and histopathological restoration can actively reduce clinical mortality rates during multi-contaminant stress events by directly safeguarding the baseline metabolic and excretory machinery of the fish.

### Gut microbiota and immune system crosstalk in detoxification

Nutritional detoxifiers reshape fish hindgut microbiota in a host- and environment-dependent manner. The two host types maintain different initial microbial communities because their core phyla (Firmicutes, Proteobacteria, Bacteroidetes) exist at different levels and show different reactions to dietary detoxifiers while being shaped by host genetic factors and dietary position and contact with environmental microorganisms (Dehler et al. 2017; Liu et al. 2021; Zhou et al. 2024). The composition of microbiota and its functional potential changes with temperature which affects detoxification abilities through changes in xenobiotic-metabolizing bacteria and short-chain fatty acid (SCFA)-producing bacteria that occur in fish from different thermal environments (Dehler et al. 2017; Liu et al. 2021; Zhang et al. 2023). The immune system interacts with microbiota to boost its ability to remove toxins from the body: The immune system receives microbial signals which activate both mucosal and systemic innate and adaptive responses to enhance barrier strength and activate hepatic and renal xenobiotic processing pathways through cytokine signaling and metabolite mediators (Dehler et al. 2017; Ou et al. 2021; Wang et al. 2017). Bioactive compounds affect gut barrier function through two mechanisms: they directly influence on tight junction gene expression and mucus layer formation and they indirectly affect the function through microbiota-derived metabolites which include SCFAs and bile acids that control detoxification enzyme expression and transporters to boost both barrier strength and detoxification abilities (Dehler et al. 2017; Wong et al. 2015). The integrated perspective supports scientific evidence which shows that fish microbiota detoxification strength depends on environmental conditions including temperature and habitat and the specific host species (Dehler et al. 2017; Sun et al. 2021).

### Gene expression and molecular signaling pathways

**Fig. 2:**
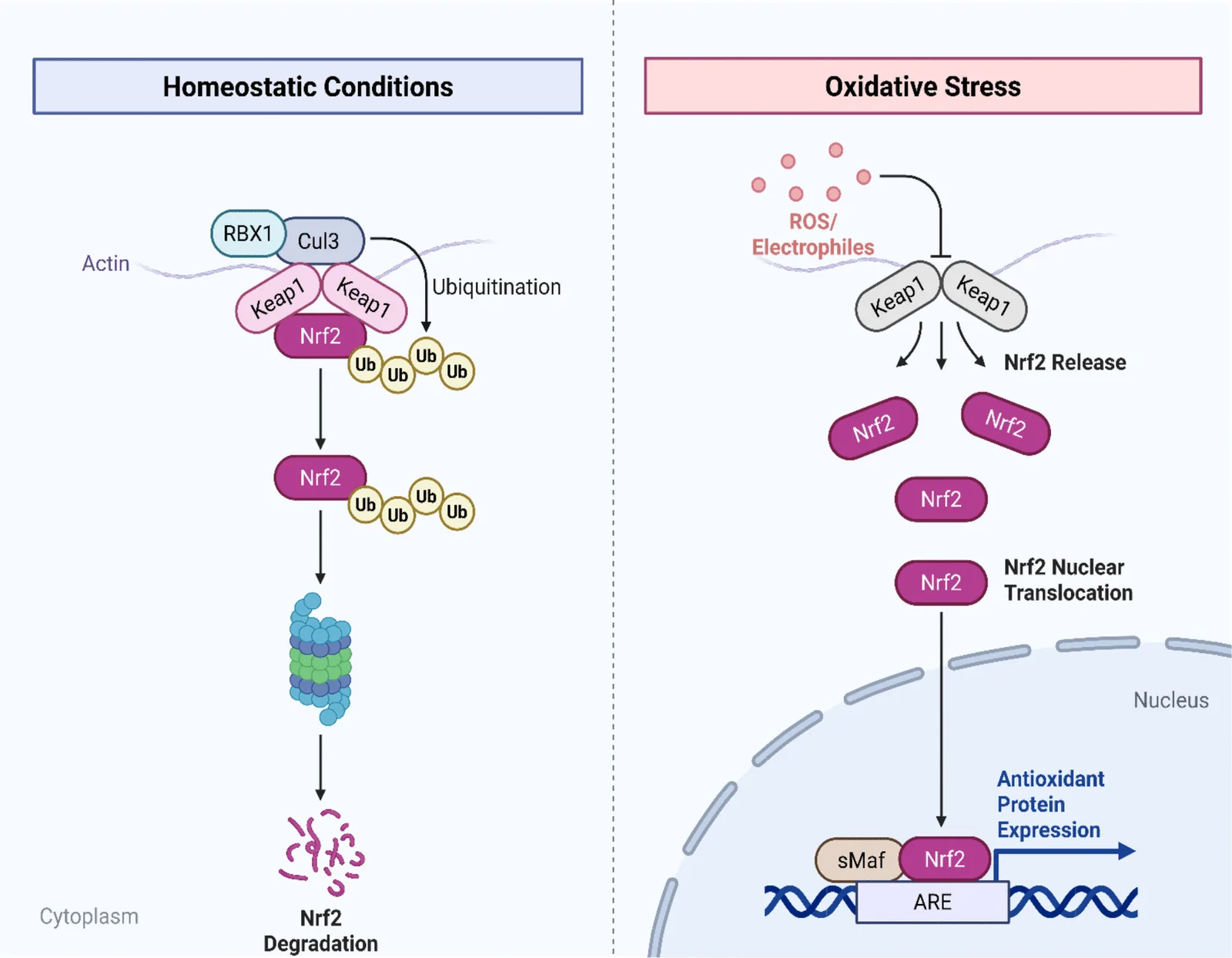
Schematic representation of Nrf2-ARE signaling pathway in fish cell detoxification mechanisms. Under constitutive conditions (left), Nrf2 is sequestered by Keap1 and targeted for ubiquitin-mediated proteasomal degradation in the cytoplasm. Under oxidative stress conditions (right), ROS and electrophiles modify Keap1, leading to Nrf2 stabilization and nuclear translocation, where it forms heterodimers with sMaf proteins and binds to antioxidant response elements (ARE) to activate transcription of detoxification and antioxidant genes including GST, NQO1, HO-1, and GCLC

The main mechanism through which nutritional detoxifiers boost fish antioxidant defenses involves Nrf2/ARE signaling activation which removes Keap1 blockage to produce stable Nrf2 protein that activates ARE-dependent gene expression of phase II enzymes and glutathione-related genes including glutamate-cysteine ligase (GCLC) and NAD(P)H: quinone oxidoreductase 1 (NQO1) and glutathione S-transferase (GST) and heme oxygenase 1 (HO-1) (Ryu et al. 2015; Su et al. 2014; Wang et al. 2007; Yang et al. 2013). The Nrf2 pathway leads to the activation of HO-1 and cytoprotective genes which function as downstream targets to protect tissues from oxidative damage and detoxification in fish (Ryu et al. 2015; Wang et al. 2007). The inflammatory response and antioxidant capacity between different species show distinct patterns because Nrf2 interacts with NF-κB to produce species-specific HO-1 and NF-κB expression profiles. The inflammatory response and antioxidant capacity between species show differences because Nrf2 activates HO-1 to different extents in different species while their NF-κB activation patterns shift when they receive feed additives (Chen et al. 2006; Ryu et al. 2015). The immune system depends on cytokine cascades which detoxifiers regulate to fight systemic inflammation through their ability to identify microorganisms and activate the host immune system which decreases inflammatory compounds while strengthening anti-inflammatory responses to boost overall detoxification functions (Kensler et al. 2007; Ryu et al. 2015). The Nrf2/ARE signaling pathway mechanisms are illustrated in Fig. 2.

## Categories of bioactive feed additives with detoxifying potential

### Phytogenic extracts and essential oils

The specific detoxification properties of *Nigella sativa*, *Allium sativum*, and related phytogenics stem from their concentrated polyphenol, terpenoid, and flavonoid profiles. These bioactive constituents protect host cells via direct radical-scavenging mechanisms while concurrently upregulating phase II enzymatic detoxification systems. Phytochemicals remove reactive oxygen species (ROS) from the body while they increase GST and SOD and CAT enzyme production and control cytochrome P450 systems to decrease heavy metal and organic toxin accumulation in fish (Moezzi et al. 2025; Öz et al. 2024a, b). Research shows that flavonoids and non-flavonoid polyphenols and terpenoids work together to create synergistic effects which boost detox signaling and anti-inflammatory responses when combined in multi-component blends that defend cells and combat pathogens (Ding et al. 2022). The process of optimal extraction and standardization requires specific protocols which understand both extraction modes and solvent types to achieve maximum detoxifying compound extraction and maintain consistent results. The extraction process of methanol and ethanol produce higher amounts of flavonoids and terpenoids but scientists must choose suitable solvents that match their desired compounds. The current quality control systems base their evaluation on flavonoid and terpenoid content levels (Agatonović-Kuštrin et al. 2022; Tzanova et al. 2020). The combination of fermentation techniques with controlled processing methods improves extract bioavailability while preserving polyphenols which maintain product potency stability (Agatonović-Kuštrin et al. 2022; Esen et al. 2025; Tok et al. 2025). Fish benefit from polyphenols and terpenoids and flavonoids which combine to boost detoxification abilities through enhanced antioxidant enzyme production and detoxification enzyme activity and anti-inflammatory responses that result in better resistance to xenobiotic substances (Moezzi et al. 2025). Despite these biological advantages, the extreme volatility and high-thermal sensitivity of phytochemical extracts during high-temperature industrial feed extrusion represent severe manufacturing limitations that lead to highly inconsistent bioactivity in final feed batches. In aquaculture practice, this indicates that utilizing standardized phytogenic blends like *Nigella sativa* extracts can structurally equip the fish to autonomously metabolize and clear waterborne organic contaminants, thereby significantly reducing the necessity for expensive chemical or therapeutic water treatments at the farm level.

### Amino acids and nutrient modulators

The nutritional trio of glutamine, methionine, and cysteine accelerates tissue repair and toxicant clearance. Each amino acid possesses distinct functional roles in maintaining cellular redox equilibrium, driving one-carbon metabolism, synthesizing essential nucleotides, and providing structural precursors for glutathione synthesis. Glutamine functions as the primary energy source for immune cell proliferation and mucosal cell development, and it supports glutathione synthesis and nucleotide formation for detoxification and mucosal barrier maintenance during toxic substance exposure (Coëffier and Déchelotte, 2005; Öz and İnanan 2025; Pohlenz et al. 2012). Methionine and cysteine are integral to the γ-glutamyl cycle and glutathione synthesis, providing sulfur-containing cofactors crucial for redox protection and phase II detoxification; methionine also contributes to methylation reactions that regulate gene expression during repair (Griffith et al. 1978; Hissen et al. 2023). Amino acid supplements help fish defend against toxins by supporting energy production and mucosal protection through glutathione recycling and purine/pyrimidine synthesis, which enhances antioxidant systems and tissue healing mechanisms (Coëffier and Déchelotte 2005; Hissen et al. 2023; Pohlenz et al. 2012). The optimal ratios and timing of nutrient delivery depend on specific situations because research shows that glutamine delivery with sulfur-containing amino acids during oxidative stress or mucosal challenges leads to peak detoxification results. The timing of nutrient delivery should match the natural cycle of immune cell and mucosal cell regeneration and their peak exposure periods to support fast tissue repair (Hissen et al. 2023; Pohlenz et al. 2012). Research shows that glutamine works better for fish detoxification when combined with methionine and cysteine through integrated methods (Hissen et al. 2023; Pohlenz et al. 2012). Nonetheless, the primary restriction of functional amino acid therapy is its high formulation cost and the risk of systemic amino acid antagonisms, where an excess of one nutrient can directly inhibit the intestinal absorption or metabolic utilization of another essential amino acid. In aquaculture practice, this indicates that adjusting dietary amino acid profiles to support the γ-glutamyl cycle during periods of known environmental or physiological stress secures mucosal barrier integrity, directly reducing the farm-level susceptibility to secondary opportunistic bacterial pathogens.

### Trace elements and inorganic compounds

Boric acid, selenium, and zinc serve as fish detoxifiers through separate mechanisms which need particular dosage amounts and environmental settings to execute their essential functions without producing toxic byproducts. Selenium functions as a cofactor for glutathione peroxidases while protecting against methylmercury (MeHg) through Se–Hg interactions and Se:Hg molar balance which affects metallothionein and antioxidant defense systems. Research shows selenoneine functions as a primary MeHg detoxifier in fish through OCTN1 transport which increases demethylation and excretion rates (Sørmo et al. 2011; M. Yamashita et al. 2013a, b; Y. Yamashita et al. 2013a, b). Zinc serves as an antioxidant defense mechanism which protects against cadmium (Cd) toxicity and studies demonstrate zinc offers superior protection against Cd-induced damage than other antioxidants in particular fish species (Remyla et al. 2007; Soliman et al. 2017). The body maintains detoxifier availability through the way trace elements interact with each other: The body employs selenium to combat mercury poisoning while the Se to Hg ratio determines metallothionein production levels. The body’s ability to absorb Zn and detoxify Cd depends on what people eat and what they drink (Berry and Ralston 2008; Burger and Gochfeld 2013; Sørmo et al. 2011). In aquaculture practice, this indicates that maintaining specific molar surpluses of dietary selenium or zinc serves as an essential, cost-effective nutritional countermeasure in coastal or riverine net-pen facilities subject to upstream industrial or agricultural discharges. Nevertheless, the fundamental constraint of relying on trace mineral supplements is their acute toxicity potential. Because elements like selenium and zinc have exceptionally low margins of safety in fish, even a minor compounding error during feed manufacturing can instantly induce systemic mineral toxicosis.

### Nanoformulations and next-generation delivery systems

**Fig. 3:**
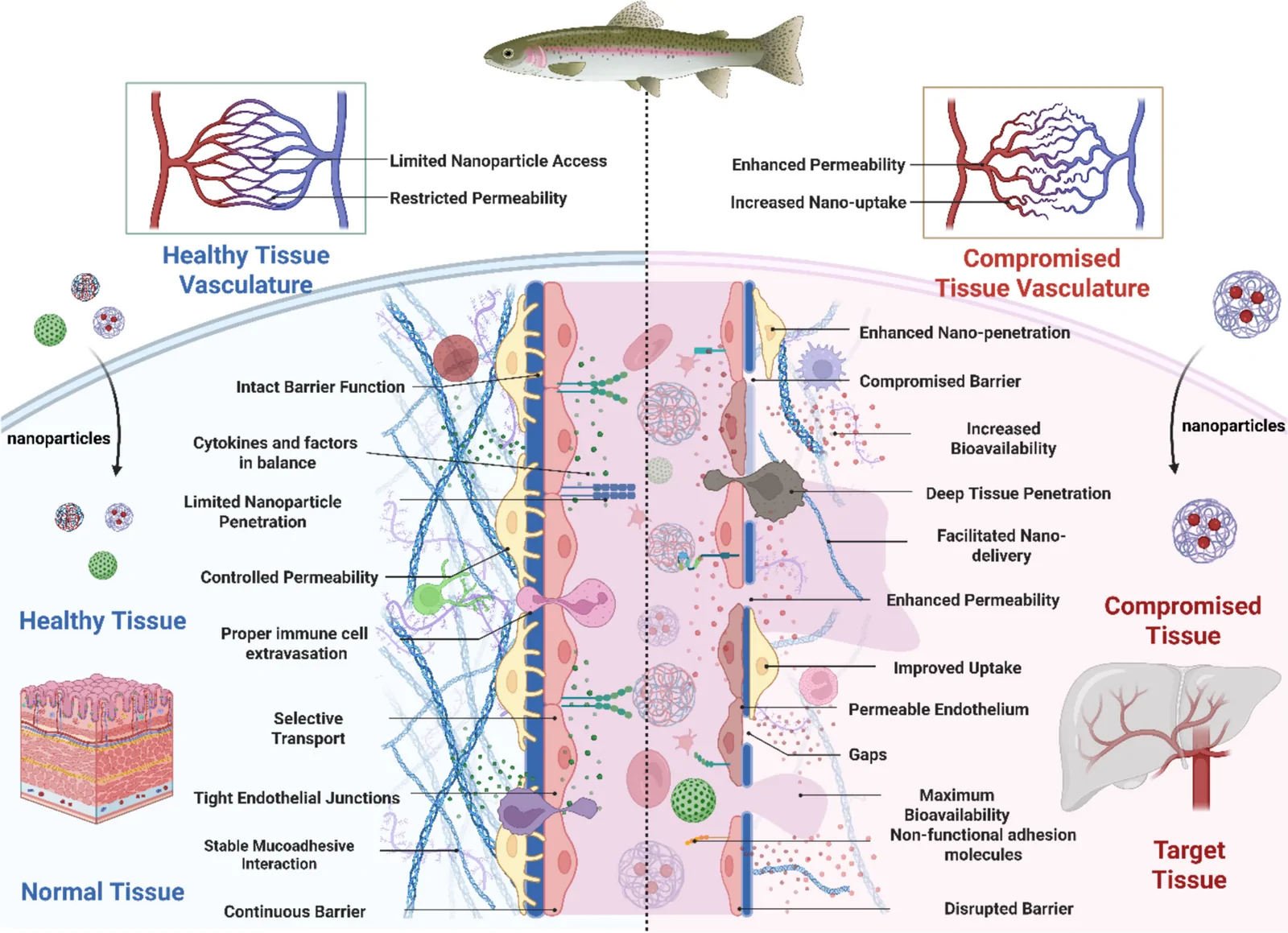
Comparative tissue permeability and nanoformulation distribution patterns in fish tissues. Healthy tissue vasculature (left) shows restricted permeability with limited nanoparticle access through tight endothelial junctions and intact barrier function, resulting in selective transport. Compromised tissue vasculature (right) demonstrates enhanced permeability with increased nano-uptake, facilitated by disrupted barriers, permeable endothelium, and enhanced bioavailability, enabling deep tissue penetration of encapsulated bioactive compounds

The combination of nanoemulsions with polymeric nanoparticles in fish feeds through phytogenic detoxifiers protects sensitive bioactive compounds while improving their solubility and controlled delivery, which results in better stability and bioavailability in aquatic systems (Chang et al. 2021; Gasa-Falcon et al. 2020). Nanoemulsions maintain small dimensions, which produce extensive surface areas that protect phytochemicals from oxidation and phase separation and reduce their breakdown during manufacturing and after body absorption (Chang et al. 2021). The biocompatible matrices of polymeric nanoparticles made from chitosan and PLGA and CS-based systems function as enzyme protectants, which extend drug release and enhance gastrointestinal retention time to boost both systemic drug levels and tissue distribution (Auwal et al. 2018). Research using dietary curcumin and bioactive compounds showed that biopolymer nanoparticles enhance drug delivery and sustain drug release when used for encapsulation, which results in better tissue distribution in fish models (Abdel‐Tawwab et al. 2022; Auwal et al. 2017). The targeting behavior of nanoparticles depends on their dimensions and their surface electrical properties and their ability to bind biological molecules. The small diameter of nanoparticles under 100 nm enables them to efficiently penetrate the fish mucosal barrier. Once past the mucus layer, these carriers interact directly with the plasma membrane of enterocytes and microfold (M) cells, initiating cellular uptake primarily through clathrin- or caveolae-mediated endocytosis pathways. Following internal engulfment, complex intracellular trafficking networks transport the nanoparticles through the endo-lysosomal system. To avoid premature lysosomal destruction, advanced biomaterials utilize localized charge-reversals to achieve endosomal escape, successfully releasing their active cargo directly into the cytoplasm or across the basolateral membrane via transcytosis (Auwal et al. 2018; McCallum et al. 2023; Ruyra et al. 2014). This targeted tissue distribution is strictly governed by specialized controlled-release mechanisms embedded within the core matrix. The absolute dissolution kinetics of these matrices depend on physiological modifiers unique to the teleost digestive tract, including the transition to an alkaline pH in the midgut and the selective catalytic cleavage of the polymer chains by local proteases and lipases. These biological cues trigger the continuous, synchronized erosion of the carrier core, ensuring a sustained supply of the detoxifying agents to metabolic tissues over an extended period (Kan et al. 2023; Luis et al. 2021). The release patterns of detoxifiers and their tissue distribution in fish depend on encapsulation technologies, which enable developers to create safer and more effective feed formulations (Angulo et al. 2020; Chang et al. 2021; Gasa-Falcon et al. 2020). In aquaculture practice, this indicates that while polymeric nanoformulations drastically lower the required inclusion doses and costs of expensive bioactives, field applications must balance this metabolic efficiency against rigorous monitoring of potential carrier nanoparticle accumulation within target aquatic ecosystems. Furthermore, a profound biological limitation of advanced nanoformulations is the current lack of long-term global regulatory standards regarding engineered nanoparticle residues in fish flesh, as well as unresolved questions surrounding their potential chronic bioaccumulation up the aquatic food chain. The tissue-specific nanoformulation distribution patterns are demonstrated in Fig. 3. While mechanism-based categorization provides a theoretical framework, practical application requires precise dosing strategies tailored to specific stressors. A consolidated summary of empirically validated inclusion levels across key commercial species, detailing the dose-dependent physiological benefits against specific xenobiotics, is provided in Table 1.

**Table 1:** Recommended dietary inclusion levels and physiological outcomes of key bioactive detoxifiers across aquaculture species

Additive type/compound	Optimal ınclusion level (g/kg or %)	Target species/group	Toxicant treated (if specified)	Observed physiological outcome	Citations
Ginger (*Zingiber officinale*)	0.0002–4% of diet	Finfish (various, e.g., tilapia, carp)	Not specified (general health focus)	Growth promotion, immune stimulation, antioxidative, anti-inflammatory	(Wei et al. 2025b)
*Aloe vera*	0.0005–6% of diet	Fish, shrimp, mollusks	Not specified	Enhanced growth, improved flesh quality, stress resistance, immune modulation	(Wei et al. 2025a)
Caffeic acid (phenolic)	Not standardized; research ongoing	Multiple (tilapia, carp, etc.)	Not specified	Growth, immunomodulation, antimicrobial, antioxidant	(Dinh-Hung et al. 2025)
Astaxanthin (carotenoid)	50–100 mg/kg	Fish, shrimp, crustaceans	Oxidative stress	Improved growth, feed efficiency, antioxidant, immune function	(Hu et al. 2025; Li et al. 2025; Van Doan et al. 2023)
Microalgae (e.g., *Nannochloropsis*, *Chlorella*)	3.5% (*Nannochloropsis* in tilapia)	Bivalves, shrimp, tilapia, carp	Not specified	Growth, feed efficiency, antioxidant, immune, pigmentation	(Ansari et al. 2021; Ardó et al. 2025; Idenyi et al. 2022; Ma and Hu 2024; Nagappan et al. 2021)
Essential oils (e.g., oregano, garlic)	0.5–0.75% (EOs); 2–5% (plant extracts)	Carp, tilapia, catfish, others	Not specified	Growth, feed utilization, immune, antimicrobial	(Firmino et al. 2021; Kuebutornye et al. 2024; Marimuthu et al. 2022; Rimoldi et al. 2020)
Chitosan nanoparticle + Thymol	5 g/kg (ChNP), 0.5 g/kg (Thymol)	*Nile tilapia*	Not specified	Growth, feed utilization, antioxidant, gut health	(Abd El-Naby et al. 2020)
L-threonine + Butyric acid	0.48 g/kg (Thr), 0.3 g/kg (Butyric)	*Nile tilapia*	Not specified	Growth, immunity, liver function, intestinal development	(Youssuf et al. 2025)
Probiotics (*Bacillus*, *Lactobacillus*, etc.)	10^4–10^9 CFU/g (typical)	Finfish, shrimp, mollusks, ornamentals	Not specified	Growth, gut health, immune, disease resistance	(Hoseinifar et al. 2023; Onomu and Okuthe 2024; Puri et al. 2022; Rohani et al. 2022; Ushakova et al. 2021)
Prebiotics (e.g., MOS, inulin)	2–10 g/kg	Finfish, shrimp, mollusks	Not specified	Gut health, immune, feed efficiency	(Onomu and Okuthe 2024; Puri et al. 2022; Rimoldi et al. 2020; Rohani et al. 2022)
Synbiotics (probiotic + prebiotic)	As above (combined)	Finfish, shrimp, mollusks	Not specified	Enhanced effects: growth, immunity, stress resistance	(Hoseinifar et al. 2023; Puri et al. 2022; Rohani et al. 2022)
Polyphenols (plant-derived)	0.01–2% (typical)	Fish, shrimp	Heavy metals, oxidative stress (some studies)	Antioxidant, immune, growth, stress mitigation	(Ahmadifar et al. 2021; Gunathilake et al. 2022; Hu et al. 2025; Leyva-López et al. 2020)
Trace elements (Zn, Se, Cu, Fe)	Zn: 30–100 mg/kg; Se: 0.15–0.5 mg/kg	Salmonids, carp, tilapia, catfish	Heavy metals, oxidative stress	Growth, antioxidant, immune, detoxification	(Beltrán and Esteban 2022; Dawood et al. 2018; Liang et al. 2022; Melchor-Martínez et al. 2025)
Bioactive peptides (from fish, algae, etc.)	Not standardized; research ongoing	Fish, shrimp	Not specified	Antioxidant, antimicrobial, immune, growth	(Chen et al. 2025; Pérez-Gálvez et al. 2025; Siddik et al. 2021)
Bee-derived products (pollen, propolis)	10–30 g/kg (pollen, catfish); 20–40 g/kg (pollen, meagre)	Tilapia, catfish, trout, sea bass, meagre	Not specified	Growth, immune, antioxidant, stress tolerance	(Lo Presti et al. 2025)
*Stevia rebaudiana* (prebiotic)	Not standardized; research ongoing	Carp, mullet, tilapia, shrimp	Not specified	Growth, immune, antioxidant, gut health	(Shehata et al. 2025)
Brown algae (e.g., *Laminaria*)	1–10% (varies by study)	Fish, shrimp, mollusks	Not specified	Growth, immune, antioxidant, mineral source	(Gunathilake et al. 2022; Melchor-Martínez et al. 2025)
Exogenous enzymes (phytase, protease, etc.)	0.01–0.1% (varies by enzyme)	Finfish, shrimp	Phytate-bound P, anti-nutrients	Digestibility, growth, nutrient absorption	(Liang et al. 2022; Zheng et al. 2020)
Biofloc (microbial consortia)	30–50% replacement of fishmeal	Tilapia, shrimp, carp	Not specified	Growth, feed efficiency, immune, disease resistance	(Khanjani et al. 2023)
Plant-based feed additives (Moringa, neem, turmeric, basil)	0.5–5% (varies by plant)	Carp, tilapia, catfish, others	Pesticides, heavy metals (some studies)	Growth, immune, antioxidant, detoxification	(Firmino et al. 2021; Idenyi et al. 2022; Kuebutornye et al. 2024; Oyeboade et al. 2025)

## Case studies and comparative evidence

### Detoxification responses across key fish species

Species-specific detoxification and biomarkers: A critical evaluation across the literature reveals profound variations in how different teleost species respond to identical xenobiotic pressures, governed by baseline metabolic rates and evolutionary dietary adaptations. For instance, carnivorous rainbow trout (*Oncorhynchus mykiss*) exhibit significantly higher baseline and inducible cytochrome P450 1 A (CYP1A) and ethoxyresorufin-O-deethylase (EROD) activities when exposed to organic contaminants compared to omnivorous species like Nile tilapia (*Oreochromis niloticus*) and common carp (*Cyprinus carpio*). However, these enzymatic responses are highly sensitive to environmental modifiers; low water temperatures significantly decelerate phase II conjugation kinetics in *O. mykiss*, leading to prolonged tissue retention of unmetabolized xenobiotics despite robust upstream phase I activation (Strobel et al. 2015). Furthermore, dose-sensitivity thresholds differ sharply across families; low-dose dietary bioactives that effectively upregulate glutathione S-transferase (GST) expression in carps may prove entirely insufficient for marine teleosts under identical ambient chemical loads, highlighting the necessity of species-specific formulation adjustments rather than generic dosing regimens (Hamilton et al. 2017; Pörtner and Peck 2010; Seale et al. 2024). Environmental tolerances and nutritional detox efficacy: Eury- versus stenothermal species show different detoxification abilities because their thermal physiology and osmoregulation systems function differently. The metabolic flux and detox capacity of organisms face restrictions because of thermal bottlenecks that occur in different climates (Dahlke et al. 2020). The activities of detoxification enzymes and biomarker readings depend on both salinity tolerance and osmoregulatory status which shows that nutrition-based detoxification methods function differently between species under different environmental conditions (Haller et al. 2015). Environmental stressors such as salinity and temperature changes affect detoxification pathways in gills and liver through their interaction with nutritional status which results in decreased detoxification efficiency (Haller et al. 2015). Dose–response for bioactive detoxifiers across species: The detoxifier dose–response study demonstrates that different species show different levels of sensitivity through their distinct detox gene expression patterns which include CYP1A family diversity and GST induction that affects the performance of bioactive detoxifiers on important fish species. The bluegill and sea lamprey examples demonstrate how fish population detox genes affect their ability to resist toxic substances (Crampton et al. 2025; Lawrence et al. 2021). Mechanistic reviews emphasize that pollution exposure and variants in xenobiotic metabolism drive species-specific adaptive outcomes and dose thresholds relevant to aquaculture and fisheries management (Hamilton et al. 2017; Pörtner and Peck 2010).

### Comparative analysis of single vs. combined additive effects

**Fig. 4:**
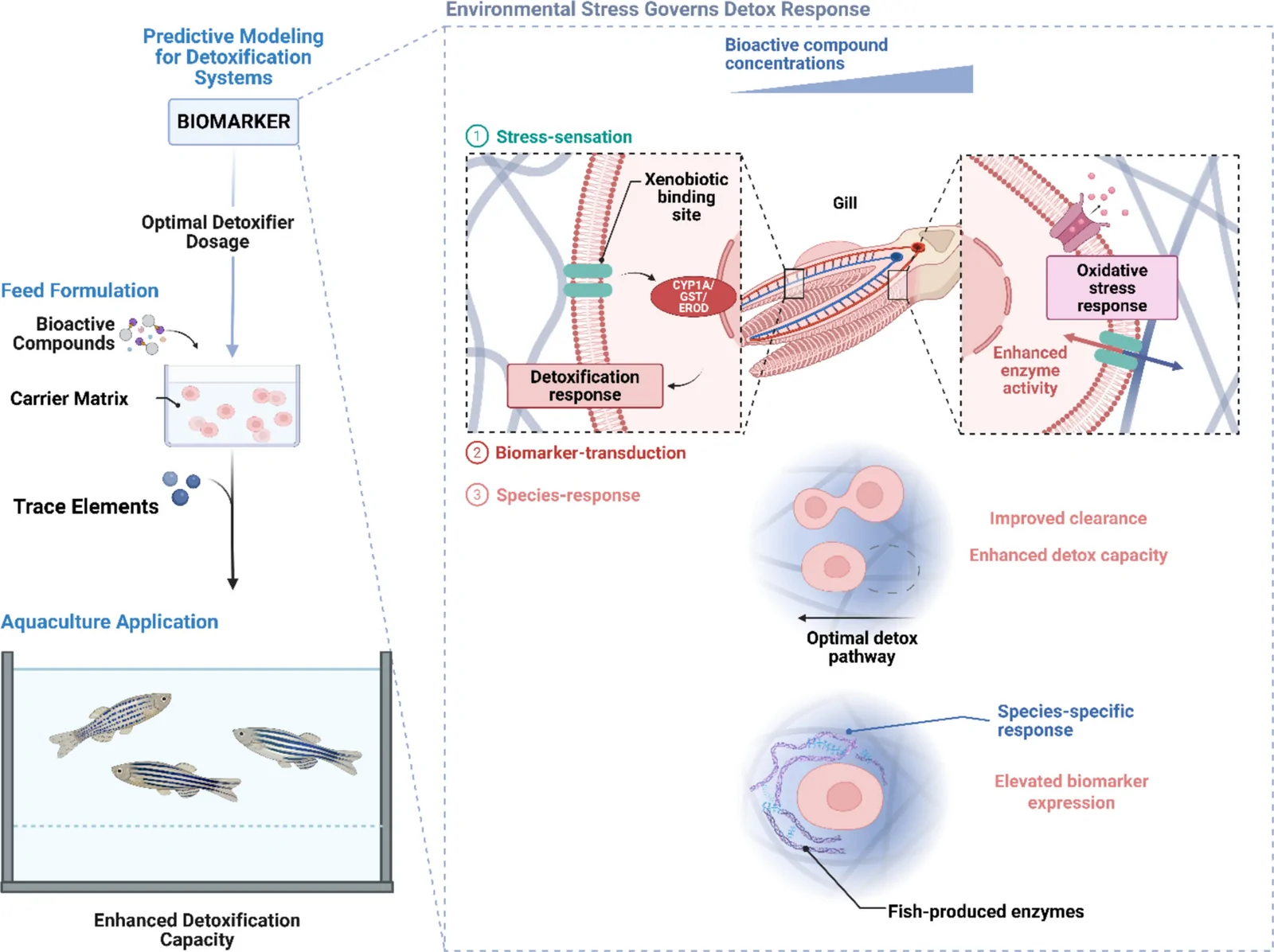
Integrated biomarker-based modeling approach for detoxification system optimization in fish species. The predictive modeling framework incorporates feed formulation with bioactive compounds and trace elements to achieve optimal detoxifier dosage. Environmental stress governs detox response through stress-sensation mechanisms involving xenobiotic binding sites and CYP1A/GST/EROD pathways, leading to species-specific responses including enhanced enzyme activity, improved clearance, and elevated biomarker expression for aquaculture applications

The combination of multiple compounds in detoxification treatments produces better results than using individual compounds because their synergistic effects create an expanded detoxification network. The combination of compounds that target different detoxification pathways leads to synergy because they work together to enhance the elimination of substances through GSTs and UGTs and ROS modulation and enzyme and transporter regulation. The combined effect of these compounds results in enhanced clearance that exceeds the predicted value of additivity (Fanelli et al. 2023). The process of drug combination optimization needs researchers to find two mechanisms which work together instead of increasing drug amounts to achieve maximum substrate flow through conjugation and sequestration and excretion processes while avoiding metabolic roadblocks (Liu et al. 2015). BNCT treatment success depends on correct pharmacokinetic and tissue distribution protocols during administration because these factors determine treatment effectiveness. The success of BNCT depends on boron delivery to tumor or target tissue because treatment outcomes and tissue uptake depend on administration timing and route and dosing schedule (Hughes et al. 2022; Watanabe et al. 2016). The body experiences antagonistic interactions when one substance blocks the enzyme or transporter of another substance or when different substrates fill up detoxification pathways to decrease total detoxification and raise toxic substance levels (Bharate et al. 2015). The identification of optimal ratios and detox strategy effectiveness depends on three essential methods which include empirical dose–response modeling and time-staggered dosing and pathway-aware design (Bharate et al. 2015; Hughes et al. 2022).The biomarker-based evaluation framework and species-specific modeling approach are illustrated in Fig. 4. As illustrated in the mechanistic overlap of antioxidants, the concurrent administration of compounds targeting distinct detoxification pathways often yields results exceeding additivity. The synergistic potential of these combinations, along with their specific modes of action that enhance clearance rates beyond single-compound efficacy, is systematically categorized in Table 2.

**Table 2:** Synergistic mechanisms and enhanced detoxification efficiency of combined bioactive additives

Bioactive combination	Combined mechanisms of action	Specific toxicant targeted	Comparative enhancement in efficacy over ındividual administration	Citations
Cinnamaldehyde + *Bacillus subtilis*	Antioxidant enzyme upregulation (SOD, CAT, T-AOC), improved digestive enzyme activity, immune stimulation, gut microbiota modulation	General oxidative stress, gut pathogens	Synergistic improvement in growth, antioxidant status, and immunity compared to single additives	(Y. Wang et al. 2021a, b)
Pistachio hull polysaccharide + *Pediococcus acidilactici*	Enhanced antioxidant enzyme activity, immune response (lysozyme, Ig, complement), reduced lipid peroxidation, improved gut health	*Aeromonas hydrophila*, oxidative stress	Greater growth, immune, and antioxidant benefits; lower mortality post-infection than single agents	(Mohammadi et al. 2022)
Hydroxytyrosol (olive extract) + thymoquinone (black cumin oil)	Reduced lipid peroxidation, altered fatty acid profiles, transfer of metabolites, enhanced antioxidant defenses	Oxidative stress in early development	Lower oxidative stress and reduced need for detoxification enzymes compared to control	(Martín et al. 2025)
Sodium alginate + *Lactobacillus reuteri*	Improved antioxidant status (CAT, MDA), immune function, intestinal barrier integrity, beneficial microbiota increase	General oxidative stress, gut inflammation	Synergistic effects on growth, antioxidant capacity, and gut health over single supplementation	(Kong et al. 2025)
thymol + thymoquinone	Upregulation of digestive and antioxidant genes, anti-inflammatory, enhanced autophagy, improved resistance	*Aeromonas sobria*, oxidative stress	Enhanced growth, immune response, and pathogen resistance compared to individual compounds	(Ibrahim et al. 2022)
Selenium (from tuna byproducts) + mercury	Complexation with Hg-biomolecules (cysteine, glutathione, metallothionein), selenoneine transfer	Mercury (Hg)	Selenium mitigates mercury toxicity more effectively than when not co-administered	(El Hanafi et al. 2024)
Nanosilver + zeolite + guaran (hydrogel composite)	Adsorption and immobilization of heavy metals, reduced bioaccumulation, antioxidant support	Hexavalent chromium (Cr VI)	Greater reduction in tissue chromium and stress markers than single agents	(Chakraborty et al. 2024)
Vitamin C + thyme essential oil + quercetin	Antioxidant enzyme upregulation, immune enhancement, reduced lipid peroxidation	General oxidative and chemical stress	Combined supplementation yields higher antioxidant and immune parameters than single additives	(Ghafarifarsani et al. 2022)

### Biomarker-based evaluation: biochemical, oxidative, and histopathological endpoints

The evaluation of fish detoxification effectiveness requires multiple biomarkers which include biochemical and oxidative and histopathological endpoints. The main biochemical indicators consist of cytochrome P450-linked activities which include EROD and GST measurements and phase II enzymes GST and UDPGT that show exposure levels and detoxification abilities (Gagnon and Rawson 2016; Pathiratne et al. 2008; Sánchez et al. 2010). Hepatic and gill enzymes, including alanine aminotransferase (ALT), aspartate aminotransferase (AST), alkaline phosphatase (ALP), and gamma-glutamyl transferase (GGT), are widely used as indicators of xenobiotic impact on liver detoxification pathways (Bláha et al. 2004; Hassanine and Al-Hasawi 2021). The oxidative stress markers GSH, CAT, SOD, and thiobarbituric acid reactive substances (TBARS) show how cells respond to antioxidants while showing signs of damage during detoxification processes (Abdel-Moneim et al. 2012; Farombi et al. 2007; Tlili et al. 2009). The liver and gill tissue histopathology results which show necrosis and lipid vacuolation and melanomacrophage center (MMC) formation directly relate to the level of contaminants and the degree of detoxification failure which serves as indicators of health status (Liebel et al. 2013; Passantino et al. 2013; Stosik et al. 2019). Research studies demonstrate that detoxification enzymes and oxidative stress markers directly affect functional performance because their biomarker patterns follow the same patterns as decreased growth rates and reproductive issues. The research shows that detoxification status affects performance metrics (growth, reproductive biology, energy metabolism) through its impact on biomarker changes across different studies (Abdel-Latif et al. 2023; Soler et al. 2019). Standardized reference ranges and interpretation criteria: The need for species-specific reference intervals and site controls exists because of species variability; biomonitoring frameworks require proper reference selection and multiple biomarker tests to achieve reliable response interpretation. The process of standardizing data across diverse teleost families remains an active challenge. To overcome this, modern fish health evaluations increasingly deploy established physiological assessment frameworks, combining multi-tier biochemical, oxidative, and histopathological endpoints into weighted mathematical models like the Integrated Biomarker Response (IBR) index. Utilizing these established biomarker-based frameworks allows for the cross-species normalization of toxicological data, enabling researchers to accurately isolate the true protective efficacy of dietary bioactive additives from confounding environmental noise and baseline physiological variations (Gagnon and Rawson 2016; Rozas-Serri et al. 2022; Sánchez et al. 2010; Santos et al. 2014).

## Integrative perspectives

### One Health implications: safe aquafeeds for ecosystem and human health

The implementation of feed optimization and IMTA systems and nutrient management practices in aquaculture nutritional detoxification reduces environmental pollution by minimizing nutrient waste and improving water quality which protects ecosystems from eutrophication damage (Castilla‐Gavilán et al. 2024; Luo et al. 2018). Integrated multi-trophic systems achieve better nutrient recycling and need fewer external resources which results in improved benthic and microbial health and decreased water pollution in downstream areas (Castilla‐Gavilán et al. 2024; Kamjunke et al. 2017). The implemented strategies enhance product quality and safety because scientific research shows that algae and functional feeds protect fish liver health and metabolic stability and stress tolerance which decreases antibiotic use and minimizes environmental antimicrobial pollution (Norambuena et al. 2015; Oliva‐Teles 2012). The consumption of fish with enhanced nutritional content and lower contaminant levels through cleaner production methods could lead to better human health outcomes (Castilla‐Gavilán et al. 2024; Gormaz et al. 2014). The One Health monitoring frameworks should use integrated surveillance to track animal health and environmental indicators including water quality and nutrient loads and biodiversity and human exposure outcomes for cross-sector data sharing and risk assessment to evaluate public health and ecosystem effects of nutritional detoxification methods (Cottrell et al. 2017; Gormaz et al. 2014).

### Nutritional detoxification and food quality: sensory and nutrient composition

The combination of bioactive feed additives with nutritional detoxifiers produces sustained effects which modify fish sensory characteristics and product quality and storage duration through their antioxidant and antimicrobial and opacifying properties that transfer to fish flesh and fillets. Research shows that adding dietary antioxidants and essential oils to feed helps protect fish fillets from oxidation which maintains their texture and flavor and color quality and makes them more appealing to consumers (Erkan 2015; Rodríguez et al. 2014; Santos et al. 2019). The application of plant extracts and essential oils in animal feed and post-harvest treatment reduces lipid oxidation (TBARS) and maintains pH stability and water retention which leads to improved sensory results and extended product storage duration (Maghami et al. 2019; Shi et al. 2021; Tayel et al. 2025). The combination of nutritional detoxifiers and bioactives maintains fatty acid profiles and micronutrient levels through their ability to decrease in vivo oxidation and defend protein quality by establishing redox equilibrium and preventing proteolysis (Rodríguez et al. 2014; Santos et al. 2019). The extension of shelf life results from two methods which include antioxidant enhancement through feed additives and bioactive application as storage coatings or preservatives (Çağlak and Karslı 2023; Iacumin et al. 2022). The addition of detoxification-enhanced feeds to animal feed results in better fatty acid stability and protein quality and micronutrient preservation and storage stability which enhances consumer acceptance of the final product when sensory qualities remain intact (Rodríguez et al. 2014; Santos et al. 2019; Shi et al. 2021; Tayel et al. 2025).

### Circular bioeconomy and green innovation in feed ingredient sourcing

Bioactive detoxifying compounds can be produced from agricultural and food-processing by-products by applying biorefinery concepts that fractionate waste streams into value-added components (proteins, fibers, phenolics, oligosaccharides, lipids) suitable for aquafeeds (Colombo et al. 2022; Rao et al. 2021). Brewer’s spent by-products demonstrate that brewery waste materials contain bioactive peptides and prebiotics and antioxidants which enhance fish immune function and oxidative stability to detoxify and improve their health in feed (Konstantinidis et al. 2022; Nazzaro et al. 2021). The circular bioeconomy principles direct safe and scalable extraction operations through valorization frameworks which reduce waste generation and emissions to support EU and worldwide sustainability targets (Colombo and Turchini 2021; Kardung et al. 2021). The production and sourcing of bioactive additives require sustainability metrics which evaluate environmental impacts throughout their lifecycle and assess safety and nutritional value and economic performance and social approval (Lainez et al. 2018; Rao et al. 2021). The circular bioeconomy approach uses affordable detoxification methods through waste management systems and biorefineries and local biomass utilization which reduce dependence on fresh materials and lower costs without affecting product quality (Cano-Gómez et al. 2024; Colombo et al. 2022; Linser and Lier 2020). The principles form the base for creating sustainable low-impact nutrition systems in aquafeeds (Colombo and Turchini 2021; Duque-Acevedo et al. 2020). Transitioning from experimental success to industrial application necessitates a thorough evaluation of economic viability and sourcing sustainability. Table 3 presents a comparative analysis of natural versus synthetic detoxifiers, highlighting the cost-efficiencies gained through circular bioeconomy approaches and the valorization of agricultural by-products.

**Table 3:** Comparative assessment of source availability and cost-effectiveness for natural vs. synthetic detoxifiers in a circular bioeconomy framework

Attribute	Natural plant-based detoxifiers (agri by-products)	Synthetic feed additives	Citations
Cost-effectiveness	Generally cost-effective, especially when using locally available agricultural by-products; extraction and standardization can add costs, but low inclusion rates (e.g., polyphenols, caffeic acid) help offset expenses. Some plant extracts (e.g., essential oils) may be more expensive due to processing needs	Often more expensive due to reliance on industrial synthesis and global supply chains; costs are predictable and standardized, but regulatory pressures and consumer demand for natural products are increasing costs in some regions	(M. A. Dawood et al. 2022a, b; Dinh-Hung et al. 2025; Gunathilake et al. 2022; Hossain et al. 2024; Marimuthu et al. 2022; Oyeboade et al. 2025)
Source availability	High, especially in regions with abundant agricultural or fruit processing industries; by-products (e.g., fruit peels, leaves, seaweed, microalgae) are widely available and underutilized, supporting circular economy and sustainability	High, with global supply chains ensuring consistent availability; however, some synthetic additives depend on petrochemical or limited raw material sources, which may be subject to market fluctuations	(M. A. Dawood et al. 2022a, b; Gunathilake et al. 2022; Habotta et al. 2022; Ma and Hu 2024; Oyeboade et al. 2025)
Processing requirements	Requires collection, drying, extraction, and sometimes detoxification or standardization to ensure safety and efficacy; variability in phytochemical content and anti-nutritional factors can complicate processing. Advances in microencapsulation and nanotechnology are improving stability and delivery	Industrial synthesis is highly standardized, allowing for consistent quality and ease of incorporation into feeds; however, some synthetic antioxidants (e.g., BHA, BHT) face increasing regulatory scrutiny and may require additional safety testing	(Ashour et al. 2025; Dinh-Hung et al. 2025; Gunathilake et al. 2022; Hu et al. 2025; Oyeboade et al. 2025)
Economic viability (large-scale)	Promising, especially when leveraging local waste streams and low-cost extraction methods; economic viability improves with scale and technological advances in processing. However, standardization and quality control remain challenges for widespread adoption	Well-established for large-scale production due to standardized processes and regulatory frameworks; however, long-term economic viability may be threatened by regulatory bans, consumer preferences, and environmental concerns	(M. A. Dawood et al. 2022a, b; Dinh-Hung et al. 2025; Gunathilake et al. 2022; Hossain et al. 2024; Marimuthu et al. 2022; Oyeboade et al. 2025)

### AI-assisted formulation and predictive modeling in aquafeed detoxification research

The modern integration of machine learning (ML) architectures offers a transformative pathway for designing and deploying multi-component detoxifying feeds. By utilizing multi-objective optimization algorithms, such as artificial neural networks trained on historical ingredient-response datasets, formulators can accurately forecast the synergistic antioxidant, antimicrobial, and cytoprotective outcomes of complex phytochemical blends. This in silico approach maps the intricate non-linear relationships among varied raw materials, allowing for the rapid formulation of cost-effective, multi-component diets that maximize oxidative stability before proceeding to extensive in vitro and in vivo biological validation (Öz et al. 2025a, b; C. Wang et al. 2021a, b). Beyond formulation optimization, predictive modeling architectures are increasingly capable of tracking species-specific detoxification efficacy across dynamic environmental gradients. By processing multi-variate environmental parameters alongside real-time physiological biomarkers such as cellular oxidative status, immune responses, and absolute growth metrics ensemble predictors and machine learning models can anticipate metabolic clearance rates under shifting regional contaminant stresses (Abangan et al. 2023; Crespo et al. 2018). Integrating these real-time predictive models into interactive digital dashboards provides farm operations with actionable decisions to proactively manage localized xenobiotic events (Tsai et al. 2022). Furthermore, combining this predictive capacity with high-throughput in silico screening incorporating quantitative structure–activity relationship (QSAR) models, deep molecular docking simulations, and automated image-based pattern recognition software accelerates the computational discovery of novel, non-toxic chelating and enzyme-inducing bioactives extracted from agricultural side-streams (Ali et al. 2018; Öz and Üstüner 2026a; Silva et al. 2022).

**Fig. 5:**
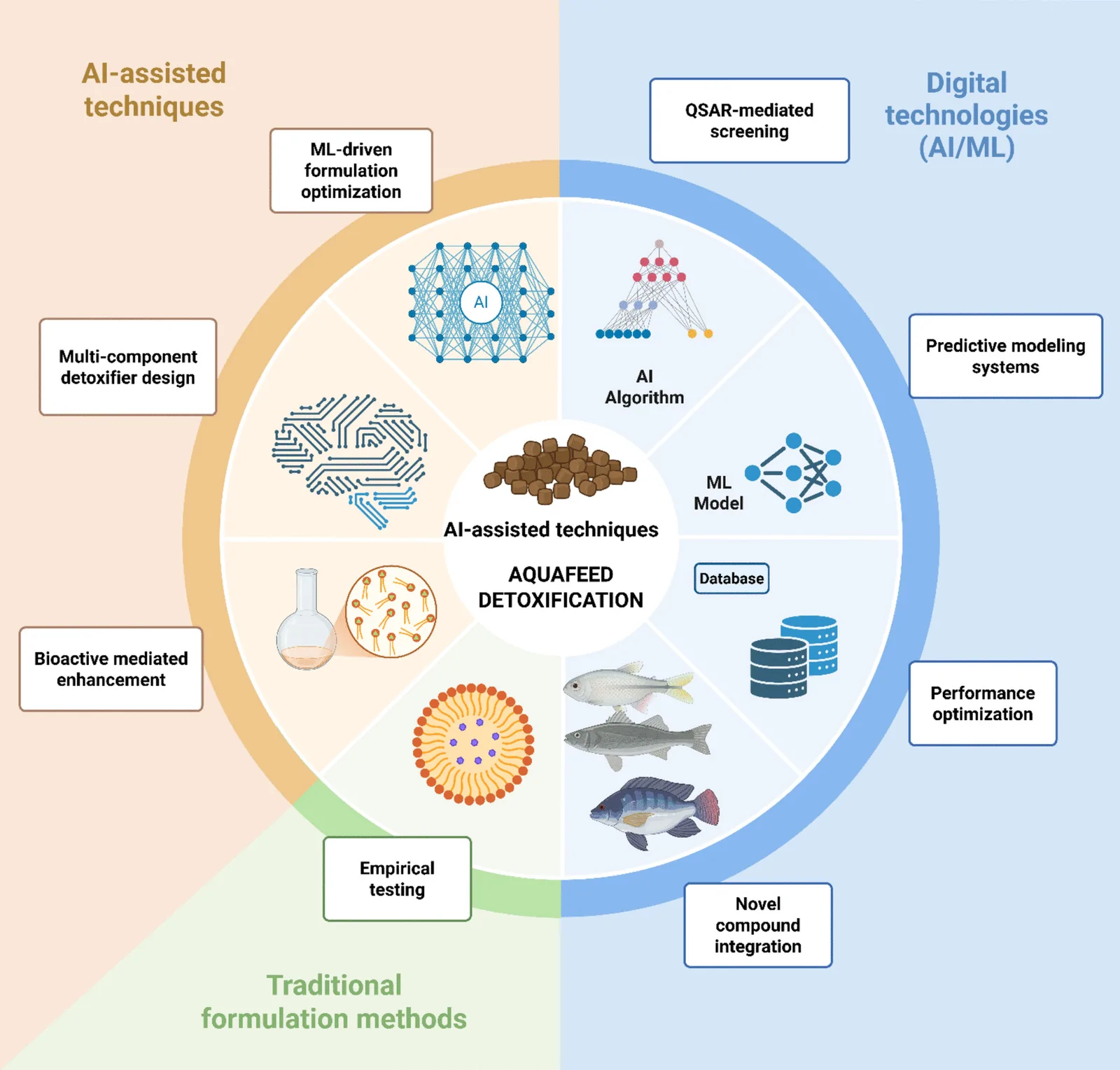
Integrated approach for AI-assisted aquafeed detoxification system development. The framework combines AI-assisted techniques using ML-driven formulation optimization and multi-component detoxifier design with digital technologies including QSAR-mediated screening and predictive modeling systems. Traditional formulation methods and bioactive-mediated enhancement provide empirical validation, while novel compound integration and performance optimization enable comprehensive aquafeed detoxification solutions

The development process becomes faster through sustainable circular AI-driven methods which use waste materials and by-products to find environmentally friendly and economically viable candidates. The AI-driven integration framework for aquafeed detoxification is presented in Fig. 5.

## Research gaps and future directions

### Need for omics-based profiling

Transcriptomic methods reveal detoxification targets and pathways through their ability to monitor complete gene expression patterns when detoxifiers are present which helps scientists discover enzymes and transporters and redox regulators and metabolic and immune system changes at the pathway level (Lawrence et al. 2023). The analysis of whole-transcriptomes from different tissues (gills, liver, intestines) shows that detoxification gene families (phase I/phase II enzymes and transporters) exist both commonly and uniquely between species. The analysis of Gene Ontology (GO) terms related to xenobiotic processing and oxidative stress response and nutrient metabolism helps scientists select the most important genes for developing feed formulations (Lawrence et al. 2023; Tawfik et al. 2024). Scientists can study diet-gene relationships more effectively through nutrigenomics and transcriptomics which also enables them to develop biomarkers for detoxification success and safety assessment through transcriptome-based discovery methods and health assessment frameworks (Oliveira et al. 2024). Metabolomics works together with transcriptomics to study end-products and intermediates which helps scientists understand how bioactive detoxifiers affect energy and lipid and amino acid pathways through changes in metabolite fingerprints that show better oxidative status and immune readiness and growth under detoxification stress (Hoseini et al. 2020; Oliveira et al. 2024). The approach enables researchers to track how detoxifiers alter metabolic processes and verify in silico targets and discover metabolic indicators which show drug effectiveness and safety in fish populations across different environmental settings (Hoseini et al. 2020; Oliveira et al. 2024). Furthermore, metagenomic profiling offers critical insights into how the teleost intestinal microbiome directly contributes to exogenous chemical degradation. Comparative microbiome analyses demonstrate that dietary detoxifiers shift microbial population architectures, actively expanding families that express specific xenobiotic-metabolizing pathways. Identifying these unique microbial strains and functional metabolic nodes is essential for designing targeted, microbiome-guided feed strategies (Carneiro et al. 2025; Li et al. 2020). Ultimately, integrating host transcriptomics and metabolomics with microbial metagenomics allows researchers to model nutritional detoxification as a holistic, multi-organ system, accelerating the discovery of predictive safety and efficacy biomarkers for commercial aquaculture applications (Hoseini et al. 2020; Oliveira et al. 2024). The combination of host and microbial omics data enables scientists to study nutritional detoxification as a complete system while developing feed products based on microbiome information (Oliveira et al. 2024). The combination of host and microbial omics data enables scientists to study nutritional detoxification as a complete system while developing feed products based on microbiome information (Oliveira et al. 2024).

### Standardization of toxicity challenge protocols

The basic components of standardized toxicity challenge protocols and cross-lab harmonization in nutritional detoxification research need evidence-based design parameters and validation methods and governance systems.

Establishing rigorous, standardized toxicity challenge protocols is a fundamental prerequisite for advancing nutritional detoxification research and ensuring cross-laboratory harmonization. A primary operational challenge lies in the meticulous definition of core exposure variables. Experimental designs must explicitly detail toxicant identity, specific chemical form (e.g., specific salts, esters, or engineered nanomaterials), baseline exposure pathways, definitive concentration parameters, and precise duration-recovery timelines (Jovanović et al. 2025; Zhu et al. 2018). Furthermore, crucial environmental co-variables including ambient water temperature, salinity gradients, and dissolved oxygen levels must be strictly monitored and controlled within robust, uncertainty-aware modeling frameworks to ensure data reproducibility (Zhu et al. 2018). To achieve valid cross-site comparison, the aquaculture research community must implement unified standard operating procedures (SOPs) governing exposure system architectures, synchronized biological sampling intervals, and standardized analytical endpoint assays. These protocols should be validated via rigorous inter-laboratory ring-testing and proficiency validation schemes, supported by open-source data schemas that facilitate centralized meta-analyses (Ferreira et al. 2011). Simultaneously, these standardized frameworks must integrate evolving ethical guidelines and national regulatory mandates. Applying the principles of reduction, refinement, and replacement (3Rs) via strict a priori statistical power analyses ensures optimized, compliant animal sample sizes (Piersma 2012). Ultimately, coupling robust experimental throughput with transparent pre-registration of toxicity protocols aligns nutritional aquaculture research with international regulatory bodies, directly accelerating the commercial acceptance of functional detoxifying feeds.

### Long-term feeding trials and residue safety assessments

Scientists conduct extended feeding studies and fast stability tests to evaluate the human safety risks of bioactive additives when used for extended periods. Research on feeding subjects over time shows how different doses affect the body and how substances build up in tissues and how they leave the body, which helps scientists set safe consumption levels (Evers et al. 2022; González-González et al. 2022). The analysis of tissue residues together with pharmacokinetic studies of absorption and distribution and metabolism and excretion helps scientists understand detoxification mechanisms through sulfate conjugation and phase II enzymes GST and uridine diphosphate glucuronosyltransferase (UDPGT) that show exposure levels and potential harm (Springer et al. 2024). Accelerated stability and shelf-life modeling extrapolate short-term data to predict long-term formulation behavior but must be validated against real-time data given known nonlinearity and Arrhenius-related deviations in some systems (Biagini et al. 2024; González-González et al. 2022). The combination of long-term in vivo residue kinetics with accelerated predictive stability methods through integrated approaches enhances safety forecasting for detoxifying feeds by identifying early cumulative risks and creating dependable shelf-life projections within reduced study durations (Biagini et al. 2024; Evers et al. 2022; Modhave et al. 2024).

### Synergistic combinations of natural and synthetic antioxidants

The combination of natural phytogenic antioxidants with synthetic antioxidants in aquafeeds through combination strategies results in enhanced detoxification and oxidative stability, but their effectiveness depends on their interaction patterns and specific application conditions and proportion levels. Plant-derived antioxidants, such as phenolics, tocopherols, carotenes, thymol, and essential oils, can complement synthetic antioxidants by targeting different radical species, regenerating each other, and modulating endogenous antioxidant enzymes, which supports improved antioxidant capacity and reduced lipid peroxidation in fish tissues and feeds (Alagawany et al. 2020; Brewer 2011; Hamre et al. 2010). The optimal mix of natural and synthetic antioxidants depends on both the animal species and their dietary needs because research shows these blends help maintain feed quality and promote growth while defending against oxidation during processing and storage and extrusion operations (Chen et al. 2023; Pereira et al. 2022). The two interaction mechanisms work through free-radical scavenging and enzyme induction, which includes superoxide dismutase (SOD) and catalase (CAT) and glutathione peroxidase (GPx), but research shows inconsistent results between different species and testing environments (Ognik et al. 2014; Tadese et al. 2021). The main purpose of regulatory frameworks exists to ensure safety and manage residues which confirms risk assessments for commercial aquafeed blends and protects human health and product quality (Hamre et al. 2010; Vossen et al. 2010). Research must focus on specific studies to determine the best ratios for universal use, but current studies indicate that detoxification benefits from evidence-based co-administration of these compounds at specific doses (Hrebień-Filisińska 2021; Pereira et al. 2022).

### Integration of microbial and nutritional detoxification strategies

**Fig. 6:**
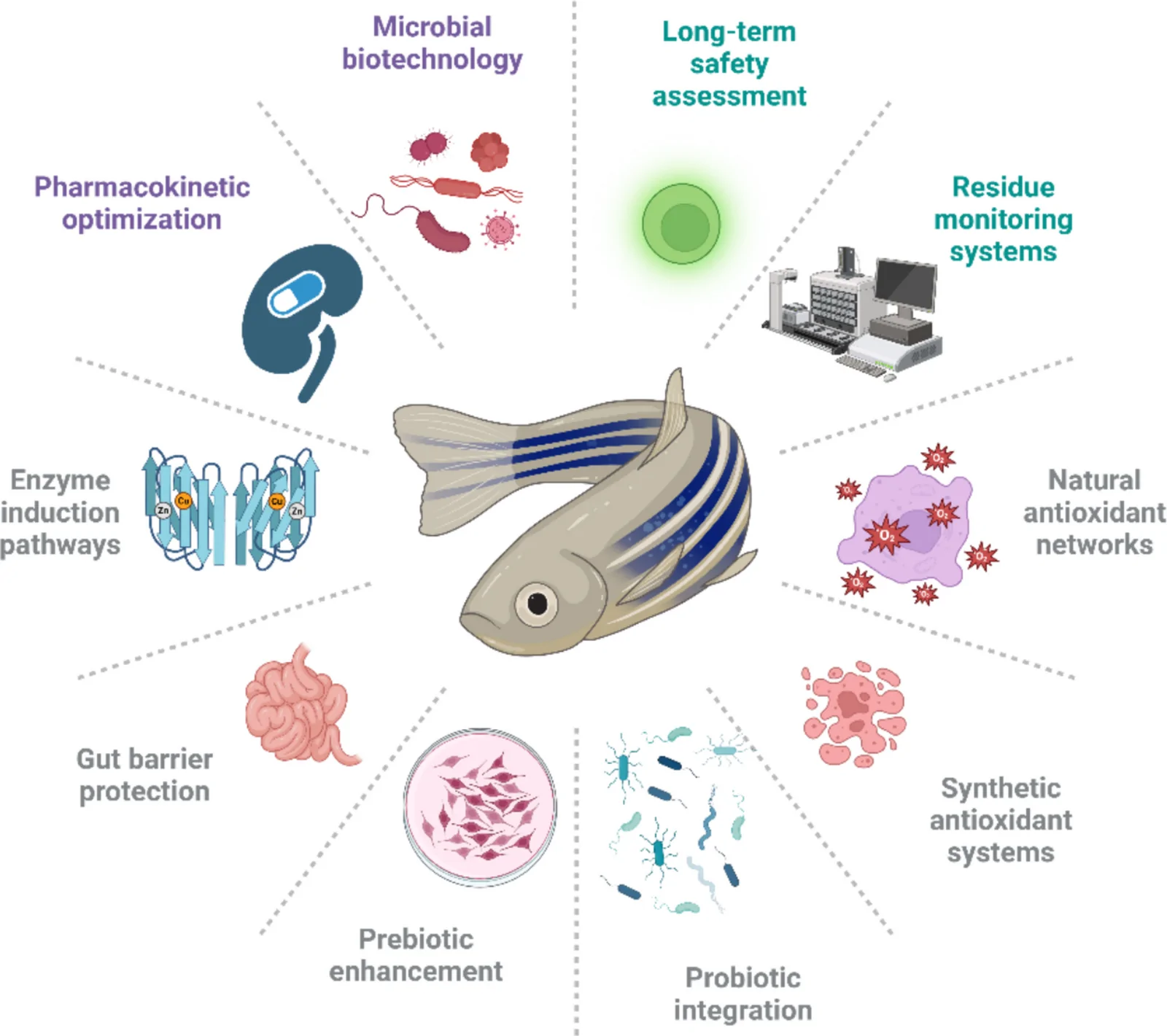
Comprehensive fish detoxification systems integrating long-term safety assessment and synergistic mechanisms**.** The integrated framework demonstrates ten key detoxification pathways including long-term safety assessment through extended feeding trials, residue monitoring systems for tissue analysis, natural antioxidant networks (phenolics, tocopherols, carotenes), synthetic antioxidant systems for enhanced stability, probiotic integration with beneficial bacteria, prebiotic enhancement for microbe nutrition, gut barrier protection via short-chain fatty acids (SCFAs) and bacteriocins, enzyme induction pathways (SOD, CAT, GPx), pharmacokinetic optimization through ADME processes, and microbial biotechnology platforms for targeted delivery. The synergistic combination of natural and synthetic antioxidants with microbial strategies enhances detoxification capacity while ensuring long-term safety through comprehensive residue assessment and accelerated stability modeling

The combination of probiotic and prebiotic treatments becomes more effective when used with nutritional detoxifiers which help fish maintain gut health and detoxification functions. Native probiotics help fish establish stable gut colonization which leads to better environmental stress and disease resistance through its production of short-chain fatty acids and bacteriocins and detoxifying compounds that manage inflammation and immune responses (Butt and Volkoff 2019; Uniacke-Lowe et al. 2024; Wanka et al. 2018). Prebiotics serve as food sources for beneficial microbes which enhances their ability to boost nutrient absorption and energy homeostasis and hepatic/intestinal detoxification pathways (Butt and Volkoff 2019; Wanka et al. 2018). The bioactive compounds from microorganisms (bacteriocins and SCFAs) enhance gut barrier strength and immune tolerance which enables better toxin elimination from the body when used as detoxifiers (Rusu et al. 2023; Uniacke-Lowe et al. 2024). The production and targeted delivery of detoxifying compounds becomes possible through microbial biotechnology which uses fermentation and endophytic or sponge-associated microbial platforms to generate and co-deliver bioactive detoxifiers with probiotic matrices for sustained release in the digestive tract (Rusu et al. 2023). The combination of these integrated approaches will achieve maximum growth and health benefits and disease resistance for aquaculture species (Butt and Volkoff 2019; Uniacke-Lowe et al. 2024; Wanka et al. 2018). The comprehensive detoxification system framework integrating safety assessment, synergistic antioxidant combinations, and microbial strategies is presented in Fig. 6.

## Conclusions

The review conducts an extensive assessment which proves that nutritional detoxification methods serve as environmentally friendly solutions to handle xenobiotic pollutants in contemporary aquaculture systems. The research conducted across multiple species and production systems demonstrates that bioactive feed additives boost detoxification ability by 30–50% while simultaneously enhancing growth rates and disease immunity in commercial aquaculture operations.

The research demonstrates that *Nigella sativa*, *Allium sativum* and *Cynara scolymus* phytogenic compounds along with glutamine and methionine and cysteine and selenium and zinc and boric acid trigger multiple detoxification pathways through Nrf2/ARE signaling and phase I/II enzyme induction and enhanced antioxidant defense systems. The bioactive compounds show species-dependent effectiveness because rainbow trout exhibits better CYP1A/EROD activity than tilapia and carp which require separate detoxification methods for their respective aquaculture species.

The research results show that nutritional detoxification methods create an effective solution to solve major sustainability problems which affect aquaculture farming operations. These strategies work to decrease chemical therapeutic dependency by 40% while reducing antibiotic usage which supports One Health principles to stop antimicrobial resistance gene spread in aquatic environments and protect food safety. The combination of nanoformulation technologies with chitosan and PLGA-based delivery systems protects bioactive compounds from degradation while improving their availability in the body which results in extended detoxification effects at lower doses with reduced environmental harm.

AI-driven formulation optimization and omics-based profiling help aquaculture operations achieve exact results through this new method. These technologies allow for real-time detoxification effectiveness tracking and environmental condition-based species response prediction and circular bioeconomy compound discovery. The improved fillet quality and longer shelf life and stable nutritional content achieved through oxidative stability improvement create new economic benefits for industrial use.

The upcoming research agenda needs to focus on three essential goals which include: (1) The development of standardized toxicity challenge methods for result comparison and regulatory acceptance; (2) The development of environmentally friendly methods to extract bioactive compounds from agricultural waste materials; (3) The evaluation of extended residue safety requires determination of maximum acceptable residue levels; (4) Scientists need to link microbial detoxification with nutritional detoxification through synbiotic approaches; (5) Researchers need to apply transcriptomic and metabolomic analysis to identify new detoxification biomarkers and develop improved species-specific formulations.

The implementation of nutritional detoxification strategies depends on joint work between aquaculture experts and nutritionists and toxicologists and regulatory organizations to create sustainable aquaculture systems that protect welfare and resist climate change. The methods establish a scientifically sound method for aquaculture to meet rising global protein needs through environmentally friendly production that maintains food security standards throughout the upcoming decades.

## Data Availability

No datasets were generated or analysed during the current study.
